# Risk factors for nodal metastasis in immunocompetent patients with low-risk squamous cell carcinoma

**DOI:** 10.1016/j.jdin.2023.04.015

**Published:** 2023-06-24

**Authors:** Kimberly A. Sable, Annika P. Weinhammer, Kyle E. Rudningen, Yaohui G. Xu

**Affiliations:** a Department of Dermatology, University of Wisconsin, Madison, Wisconsin; b CentraCare Dermatology, St. Cloud, Minnesota

**Keywords:** low-risk squamous cell carcinoma, nodal metastasis, nonmelanoma skin cancer, risk factors

*To the Editor:* While there are considerable data assessing nodal metastasis and high-risk cutaneous squamous cell carcinoma (cSCC), similar data evaluating low-risk cSCC and nodal metastasis is limited.^1^, ^2^ We conducted a retrospective review to identify immunocompetent patients with low-risk cSCC subsequently developing nodal metastasis to better characterize patient and tumor risk factors to aid clinicians in their management of this patient population. Retrospective chart review was performed at the University of Wisconsin, retrieving 2 cases over a 10-year period (Fig 1). Three additional patients were added to the study cohort who were identified outside of this data retrieval period. Patients with a diagnosis of cSCC and lymph node sampling were identified utilizing relevant ICD-9/10 and CPT codes. Patients with low-risk cSCC that subsequently developed positive regional lymph node metastasis were included for analysis. Low-risk cSCC was defined as being stage T1 or T2a by the Brigham and Woman’s Hospital (BWH) classification system. Patients with negative lymph node sampling, immunocompromised state, or non-cSCC tumors were eliminated.

**Fig 1:**
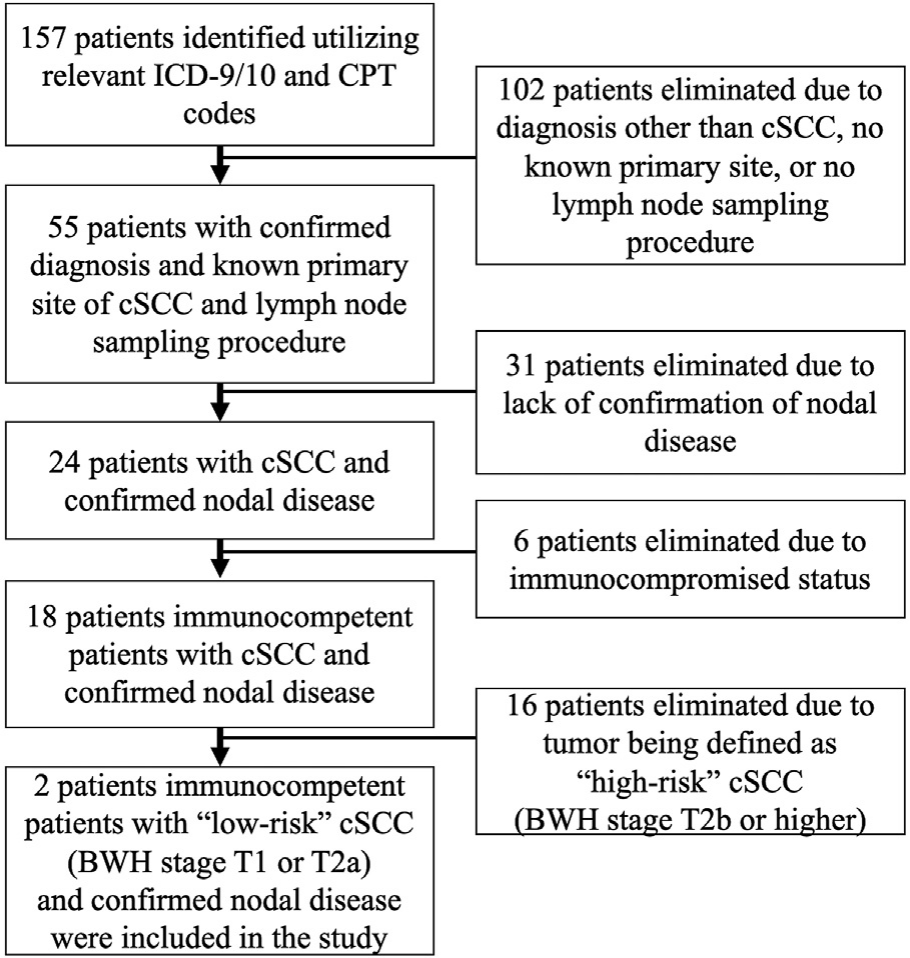
Flow chart diagram reporting methodology for identifying cases obtained over a 10-year data retrieval period (July 1, 2011 - June 30, 2021). Three additional patients were included for analysis that was obtained outside of the data retrieval period.

A total of 5 immunocompetent patients with low-risk cSCC and subsequent node metastasis were identified. The average age was 70 (range 39-90) and 3 patients were male (Table I). All patients with reported race/ethnicity were Caucasian and non-Hispanic/non-Latino. One patient’s race and ethnicity were unavailable. Tobacco use was noted in 3 patients and 3 patients had a history of alcohol use.

**Table I:** Summary of patient demographic and tumor specific data

Variable	Number (*N* = 5)
Gender	
Male	3 (60)
Female	2 (40)
Age	70 (39-90)
Race	
Caucasian, non-hispanic, non-latino	4 (80)
Unavailable	1 (20)
History of smoking	3 (60)
History of alcohol use	3 (60)
History of prior radiation	0 (0)
Location	
Head/Neck∗	4 (80)
Extremity	1 (20)
Trunk	0 (0)
Tumor size	
1-2 cm	2 (40)
2-3 cm	1 (20)
3-4 cm	2 (40)
Primary method of treatment	
Surgical excision	2 (40)
Mohs surgery	1 (20)
Electrodessication and curettage	1 (20)
Excisional biopsy	1 (20)
Tumor staging	
T1	4 (80)
T2a	1 (20)
Tumor differentiation	
Well-differentiated	3 (60)
Moderately-differentiated	1 (20)
Poorly-differentiated	0 (0)
No differentiation specified	1 (20)
Nodal metastasis location	
Cervical lymph nodes	4 (80)
Inguinal lymph nodes	1 (20)
Metastasis identification method	
Fine needle aspiration	4 (80)
Selective lymph node dissection	1 (20)
Metastasis suspected clinically	5 (100)
Imaging modality to diagnose metastasis	
Computerized tomography	4 (80)
Ultrasound	1 (20)
Imaging positive for nodal metastases	5 (100)
Time from skin cancer diagnosis to nodal metastasis identification	189 d (29-556)

Results are presented as frequencies with percentages in parentheses, or with the mean and range in parentheses.

∗ Specific location of tumors localized to the head/neck region include the right neck, right angle of the jaw, left neck, and left cheek.

A total of 4 tumors were located on the head/neck. Initial tumors were treated with Mohs surgery (2 tumors), surgical excision (1 tumor), electrodessication and curettage (1 tumor), or excision biopsy (1 tumor). In terms of staging, 4 tumors were stage T1, and 1 was stage T2a utilizing the BWH staging criteria. Three tumors were well-differentiated, 1 was moderately differentiated, and 1 did not specify differentiation. Tumor size ranged from 1.2-4 cm. No tumors had perineural or lymphovascular invasion.

In terms of nodal metastasis, 4 patients had metastases to the cervical region and one to the groin. Metastases were suspected clinically for all patients and confirmed by fine needle aspiration in 4 patients. All patients underwent imaging, with 4 utilizing computerized tomography. Imaging was positive for nodal metastasis in all patients. The average time from skin cancer diagnosis to nodal metastasis identification was 189 days (range 29-556).

In our cohort, the majority of patients with low-risk cSCC developing nodal metastasis were male, above age 65, Caucasian and non-Hispanic/non-Latino, and with history of tobacco or alcohol use. These findings suggest patient demographics including race/ethnicity, age, gender, and substance use may be considered when managing low-risk cSCC. Further analysis of this specific cohort of patients should be considered with additional large-scale studies.

Study limitations include sample size due to the infrequency of low-risk cSCC tumors undergoing metastasis. Future research direction includes expansion of our patient cohort, prospective study design, and obtaining nodal metastasis tissue for further analysis of tumor specific characteristics. Given the significant morbidity of a patient developing nodal metastasis from a low-risk skin cancer, further mechanistic studies are warranted, and cross institutional collaboration should be considered.

## Conflicts of interest

None disclosed.
